# Conscious active inference I: A quantum model naturally implements the path integral needed for real-time planning and control

**DOI:** 10.1016/j.csbj.2025.09.017

**Published:** 2025-09-13

**Authors:** Michael C. Wiest, Arjan Singh Puniani

**Affiliations:** a Neuroscience Department Wellesley College, 21 Wellesley College Rd., Wellesley, MA 02481, United States; b University of Pittsburgh Rehab Neural Engineering Labs, 1622 Locust St, 4th Floor, Pittsburgh, PA 15219, United States

**Keywords:** Microtubules, Orch OR, Predictive coding, Active inference, Consciousness, Optimal control, Bayesian brain

## Abstract

Active inference is a general framework for optimizing the behavior and learning of a sentient agential system. It may be interpreted as a general theory of sentient behavior and has been used to quantitatively model a wide variety of perceptual and behavioral contexts. Moreover, variables in neural process models of active inference appear to be represented by specific pathways in the brain, and they predict some features of actual neural responses and behavioral patterns in a variety of contexts. These applications support the validity of the active inference framework for describing real animals. However, implementing active inference in a conscious agent requires a system capable of sophisticated probabilistic computations, including a weighted average over its potential future trajectories—a path integral. Although it is straightforward to construct realistic classical biophysical neural models to approximate these computations in simple contexts, we argue in this first of two companion papers that classical Hodgkin-Huxley-style neurons are unlikely to be capable of performing these computations *quickly enough* in a realistic context. We then explain that conscious (temporally deep) active inference is mathematically equivalent to the path integral that underlies quantum dynamics. A quantum model thus provides a natural, biologically plausible mechanistic implementation of the processing required by active inference. In the second paper we review independent strong theoretical and experimental evidence from my (Wiest) lab and others’ supporting the “Orch OR” quantum theory of consciousness as a collective quantum property of intraneuronal microtubules, which explains the existence of discrete cycles of perceptual inference.

## Graphical Abstract

**Figure:**
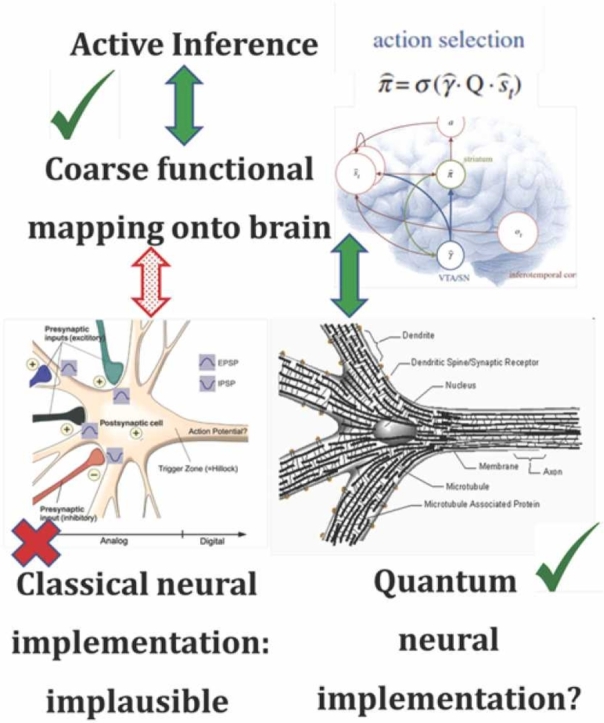

## 1 Introduction: active inference is a unified theory of sentient behavior

Active inference is a theory of sentient behavior that *unifies* optimal inference and optimal control. In active inference [100], [48], you are your beliefs about the world and beliefs about how best to act. Your beliefs are distributions representing probabilities of potential actions, possible “hidden states” or causes in the world, and possible observable outcomes of those hypothetical causes. These beliefs are grounded in a model of the world. So really, you are the model that entails, or *generates*, your beliefs.

As we will explain further below, the model evolves so as to minimize the “surprise” of unexpected *and undesirable* outcomes. Minimizing surprise is equivalent to maximizing “evidence” for our beliefs, our model—which again, is each of us. This is why organisms in active inference are said to be “self-evidencing” [73].

A process of inference selects approximately optimal beliefs about the world and approximately optimal actions based on those beliefs. What distinguishes conscious (aka sentient) agents from unconscious processes is an ability to incorporate possible futures—plans—into the moment-to-moment optimization of our beliefs and behavior. This may be quantified in the theory as “temporal depth” or “counterfactual depth” representing the length of time and number of possible trajectories that are available to the agent for consideration [100], [42].

Active inference is a *normative theory* of optimal inference and behavior [100], meaning that it prescribes what an agent *should* believe and do—to the extent that it can. Specific *process theories* [100], [46] propose hypothetical mappings of variables and computations in the model onto specific neural populations or pathways, and these process theories can predict the form that neural responses should take in the various anatomical modules. The process theories take for granted mechanisms that will account for how real neurons can flexibly add and multiply their activities, and other computations needed to implement active inference. Therefore, to approach a complete understanding of brain function, process models must be supplemented with *mechanistic models* that account for how realistic neurons could *implement* the requisite computations [110], [85].

In what follows we will briefly review the cycle of computations required for conscious agents to perform active inference, along with the literature on the relevant neural mechanisms. We will find reasons to doubt that realistic classical neural models can perform the requisite computations *fast enough* for real-time perceptual inference on sub-second time scales. Having described implementation challenges these classical models must address, we will introduce a different class of model (i.e., quantum) that is experimentally supported and may provide a more biophysically plausible implementation of real-time biological active inference in networks of real neurons.

## 2 Active inference process models impressively match neuroanatomy, physiology, and behavior

Active inference is a further development of the stochastic predictive coding framework [112], [21], [71]. Probabilistic neural units at each level of a processing hierarchy receive inputs (sensory data, observations, outcomes) which are compared to predictions from a higher level to calculate *prediction error* signals. The predictions are derived from a *generative model* comprising probability distributions that represent the agent’s beliefs about how sensory data are induced by specific *causes* (e.g., objects) in the environment, as well as *prior* beliefs about the distribution (and, in active inference, *desirability)* of the various causes. Formally, the generative model is “inverted” using Bayes’ Theorem (Eq. 1 below), to derive the *recognition model*, which is the posterior distribution over causes, updated by the latest sensory inputs. The recognition model quantifies the *responsibility* of each potential cause for the most recent observation [20](p365). For example, “that shape is probably my grandmother.” That is why it is called the recognition model.

**Fig. 1:**
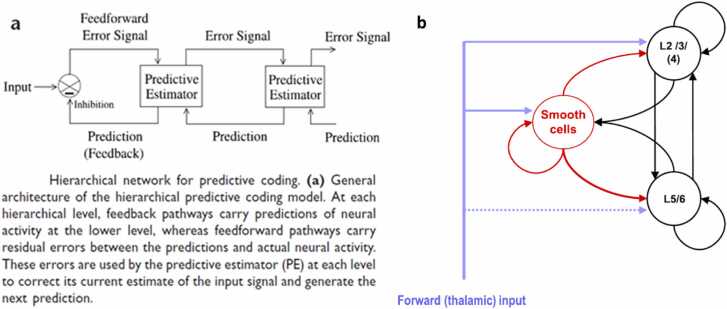
(a) Predictive coding can be understood as minimizing *prediction errors*, generated by subtracting model predictions from sensory inputs. (b) Simplified cortical microcircuit. *Black lines and arrows* depict feedback inhibitory projections representing predicted inputs, which are subtracted from the true inputs (*purple arrows*) to generate feedforward prediction error signals (*red arrows*). In *probabilistic* predictive coding, “prediction errors”. generalize to differences between whole distributions rather than between scalar values. (a) reproduced with permission from Rao et al., Nature Neuroscience, Springer Nature © 1999. This material is not covered by the article’s Creative Commons license. (b) reproduced from Bastos, A.M., Usrey, W.M., Adams, R.A., Mangun, G.R., Fries, P., & Friston, K.J. (2012). *Canonical Microcircuits for Predictive Coding*. Neuron, 76(4), 695–711. © 2012 Elsevier. Reproduced with permission.

This scheme is mapped onto neuroanatomy by identifying specific neural populations as prediction units or prediction error units, as shown in Fig. 1. Such hypothetical mappings are termed neural *process models* in active inference.

The circuits shown in Fig. 1 can be interpreted as depicting higher-level prediction units whose activities encode the most likely *values* of some represented variable (the position of a target object, for example). The descending prediction activity is subtracted from the incoming sensory input to compute a prediction error, which is fed forward to update or correct the prediction for the next cycle.

We make the transition to probabilistic predictive coding by interpreting the neural activities in Fig. 1 as somehow representing whole probability *distributions* of possible values rather than single specific values. The most common approach assumes that neural activities represent the most likely (or mean) values of constants characterizing a parametric distribution, like the mean and variance of a normal distribution. The dynamics in such a model are deterministic, though some stochasticity may be added to approximate the spiking behavior of real neurons or the unreliability of synaptic transmission. We will refer to these types of models as *deterministic* or *parametric*. An alternative approach assumes that neural activities represent a full empirical distribution by directly sampling from that distribution to generate activity patterns. These models are inherently stochastic. We will refer to them as *stochastic sampling* models. We will consider deterministic and sampling approaches in turn below.

Given one of these neural representations of probability distributions (i.e., beliefs), we further interpret the neural dynamics as implementing a cycle of Bayesian inference. In step 1 of the algorithm, the predictive population sends its representation of the *prior* distribution over states of the world-model P(s), representing beliefs about various possible values of the represented variable (e.g., target position). In step 2, the prior is compared with the input distribution via Bayes’ rule to generate an updated *posterior* belief distribution P(s|o) based on the mismatch (“prediction error”) between prior and observed input o. This posterior then acts as the prior for the next cycle of inference [20].

(1) $$P\left(s|,o\right)=\frac{P\left(s\right)P\left(o|,s\right)}{P\left(o\right)}$$

Here P(o|s) is the subject’s model of the likelihood of observation o given world-state s. Note that o and s are vectors, because they each comprise multiple scalar variables, such as different sensory inputs or distinct causes in the world-model.

Since P(o) is the probability of the observations under the subject’s world-model, it may be interpreted as the strength of *evidence* for that model. This is precisely what Bayesian statistics [51], [86] does: it uses the ratio of this evidence term between two models, known as the Bayes factor, to compare how well each model explains the data [78]. Over time, this inference cycle maximizes the evidence for the subject’s world-model. As we noted above, this “self-evidencing” evolution is equivalent to minimizing *surprise*.

The momentous step from predictive coding to the more general “predictive processing” of active inference—from *subjects* to *agents*—is to include motor (action) control states among the causes of the agent’s generative model (i.e., as a subset of the world-state variables s). At the same time, one introduces an optimistic bias into the prior beliefs about the most likely states of world (the causes), such that desirable outcomes are favored in the sense of being more probable. This step makes the model predict more favorable outcomes. The optimistic prophecies are self-fulfilling (within limits) because the model predicts favorable control states—which *enact* favorable actions via coupling between the neural control states and muscles [100].

**Fig. 2:**
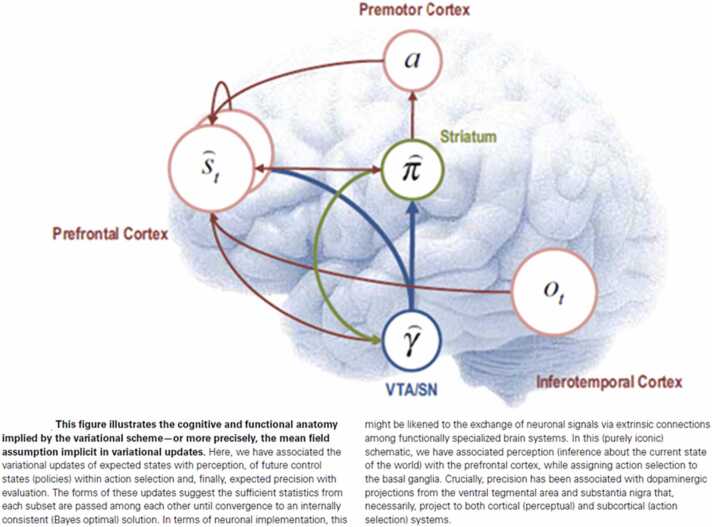
Schematic mapping of active inference onto neuroanatomy. The variational and mean-field approximations mentioned in the figure are discussed in Section 3 of the main text. The hat notation indicates a natural logarithm of the hatted probability distribution. Figure reproduced from Friston et al. [54], Frontiers in Human Neuroscience, under the terms of the Creative Commons Attribution License (CC BY).

Fig. 2 schematically depicts an agent whose modelled control states include past and future states: plans, also known as policies (π). Such an agent is said to have temporal or counterfactual depth because its belief updates consider an extended duration of “counterfactual” i.e., fictive potential action, rather than a single time step. Following [121], [125], [42], we identify conscious agents in active inference as agents with counterfactual and temporal depth, meaning they implement rapid simulations of possible futures (and pasts) to select a plan, on perceptual time scales on the order of a few hundred milliseconds. Alternative conceptions of consciousness under the active inference framework are considered in [121], [125], [36].

In each cycle of conscious active inference, sequences of observed outcomes (o) must be “explained” by sequences of causes (s) to calculate an updated plan (π) to be implemented by a sequence of actions (a). In Fig. 2, γ represents the *precision* (inverse variance) or *confidence* of the model’s predictions, which is critical for determining an optimal prediction and response. Under the active inference theory, conscious agents implement a *discrete cycle* of integrating observations and predictions over time, culminating in a discrete decision about how to update the agent’s beliefs and plans. Specific process theories have hypothesized that this perceptual cycle may correspond to theta or alpha EEG rhythms in the brain [2], [43], [95].

### 2.1 Anatomical match

Fig. 1 illustrates a first level of apparent correspondence between cortical circuitry and the basic operations of predictive processing. The rough correspondence depicted in Fig. 1 can be made more complex and realistic as in [9], which shows a more detailed correspondence between cortical anatomy and the variables of a hierarchical active inference model, while also discussing some discrepancies.

Fig. 2 illustrates a simplified schematic mapping of active inference variables onto anatomy at the level of cortical areas, from [54]. Observations, aka outcomes (o), are understood to be generated by sensory areas like visual cortex. The generative model that predicts outcomes in terms of hypothetical states (s) and causes is mapped onto frontal areas but might include other areas like medial and inferior temporal cortex and hippocampus. The subcortical motor loop (“striatum”) is understood to simulate possible plans (π), i.e., behavioral trajectories, and motor cortex implements an optimal policy via muscle activations (a for action). In active inference, *precision* of (i.e., confidence in) the agent’s predictions and chosen plans is an important variable because it determines the shape of the distribution over plans.

In Fig. 2, the dopaminergic system computes and communicates this variable to the other active inference modules that require it for their computations. This coarse match between the theory and the anatomy does not by itself constitute strong evidence that the brain implements active inference. Rather, it is consistent with that possibility. Similarly, the hierarchical process models that account for behavioral sensitivity to factors operating on multiple time scales, appear to map naturally onto the hierarchical architecture of much of the cortex [106], [111].

### 2.2 Pharmacological match

At a more detailed level than Fig. 2, other neuromodulatory systems have been suggested to signal confidence in (precision of) other specific predictions in the generative model. Specifically, dopamine is the precision of policies (as above)[113], [55], [54], [88], acetylcholine is the precision of the likelihood model’s predictions [122], [88], and noradrenaline is the precision of the dynamics in the generative model (i.e., state transitions over time)[88], [96]. Each of these functional assignments is supported by specific anatomical and physiological features of each neuromodulator system.

### 2.3 Behavioral match

Process models using such mappings can account for a variety of behaviors, including tradeoffs between exploratory and exploitative behaviors [100], [52]. The framework has been applied to model oculomotor behavior in a number of simple contexts [45], [95], [98], and has been suggested as a general theory of attention [91], [97]. It can also describe “active listening” [53], imitation behavior [111], learning and motivated behavior in general, including timing [99], [47], [49], as well as homeostatic regulation [106], [119].

### 2.4 Neural responses match and pathological symptoms match

Several process models are able to predict neuronal activity in specific stimulus contexts [99], [30], [41], [48], [92] or accompanying specific behaviors [99], [103], [105], [2], [47], [46], [50], [96], [95]. These kinds of mappings between active inference process models and neurophysiology are further supported by their ability to explain psychopathological conditions such as delusions, depression, psychosis, and others [19], [31], [49], [8], as well as motor pathologies like Parkinson’s disease [99].

Further discussion of the match between neurophysiology and active inference process models may be found in [100], [104]. From our brief review, we conclude that varied anatomical, behavioral, and neurophysiological evidence supports that the brain may implement active inference. Nevertheless, even for detailed microcircuit models like [9] or detailed process models like [46], we consider the match we have outlined between the active inference framework and the brain to be *coarse,* in that these models don’t provide *cellular mechanisms* for the computations that their neurons are assumed to carry out in order to implement active inference. We begin to consider these neural mechanisms in the next Section.

## 3 A representative process model

The active inference framework starts from the optimal process of updating beliefs according to Bayesian probability theory. However, the model inversion outlined above (Eq. 1) is intractable for most realistic generative models. Therefore, the active inference framework, following the methods developed for predictive coding, introduces a series of approximations to make the recognition and belief updating process more computationally tractable and biologically plausible (Table 1, rows a and e).

**Table 1:** Computations required for implementing real-time temporally-deep active inference. Weighted linear summation of neural activity patterns (*row b, dark pink*) are a key requirement. *Rows c, d, and h; light pink shading:* Other operations requiring linear summation, and contributions to a potential combinatorial explosion. The mean-field approximation that justifies factorizing probabilities (*row e, green*) into products (row f) is also an important part of our argument. Stochasticity (*row b, yellow*) is needed to match neural data (for deterministic models) or find optimal activity patterns (for sampling models). See text for further explanation.

We first consider the approximations and computational challenges for the deterministic parametric type of models, and then separately consider challenges for the stochastic sampling approach. Recall that in the deterministic approach to active inference [100], [46], [54], neural populations represent probability distributions by collectively estimating optimal parameter values that characterize the relevant distributions. These parameter values are the “sufficient statistics,” for example the means and variances of Gaussians in the simplest case.

Table 1 shows typical approximations adopted in neural process models, as well as critical computations required to implement the cycle of inference. We will review them each briefly before focusing on the problems we see with proposed neural implementations of linear summation of probability distributions (row b) and accounting for the observed stochasticity of neural activities (row i).

The first approximation is to introduce a variational approach that replaces exact inference with optimization based on a cost function called variational free energy (F). The next step is to adopt a familiar “biologically plausible” delta-rule for gradient descent on F (Table 1 row a). The meaning of “biologically plausible” varies with context. Here it refers to the *locality* of the synaptic plasticity rule, which means that signals needed for the plasticity algorithm are in principle available in physiological variables local to that synapse.

The cost function may be understood as an “energy” in the sense that higher energy states—i.e. beliefs and actions—are improbable; lower energies are *favored*. Again, the purpose of this is to calculate an updated belief about the environment and useful actions when it is not possible to derive an exact solution according to Bayes’ Theorem. Gradient descent will find a local minimum of the free energy, but is not guaranteed to find the global optimum. Changes in the generative model parameters that reduce F are discovered by adjusting synaptic weights by a Hebbian-like delta-rule, according to whether F just increased or decreased.

A full mathematical exposition of active inference [100] is beyond the scope of this article, but for concreteness we will review the equations describing perceptual inference and planning at each time step in a discrete process model. Using bold-font **s**_πt_ as shorthand for the estimated p(s|o) under a particular policy, at a particular time, and bold-font **o** as p(o) at a particular time; the perceptual updating rule derived from this procedure takes the following form [46]:

(2) **s**_πt_= σ(ln(**A**)·o_t_+ln(**B**_πt_)·**s**_πt-1_+ln(**B**_πt_)·**s**_πt+1_)

The matrix **A** encodes the agent’s beliefs regarding the likelihood P(o|s), of outcomes given states of the world, and **B**_πt_ is a transition matrix. **B**_πt_ encodes the agent’s beliefs regarding how hidden states including the agent’s own actions will affect the world over time.

How are these mathematical variables related to actual measures of neural activity? “This process theory associates the expected probability of a state with the probability of a neuron (or population) firing and the logarithm of this probability with postsynaptic membrane potential” [46]. In other words, the sum of logarithmic factors is assumed to be a membrane potential, with the log probability matrices representing synaptic weights from afferent connections, and the probability vectors representing the presynaptic afferent firing rates.

This is an example of the need for weighted linear sums of distributed neurons’ firing rates for implementing active inference with neurons according to this scheme.

The sigmoid function σ() mathematically translates membrane potentials to firing rates. Its biological plausibility is supported by relatively sophisticated single-neuron modeling [87]. Nevertheless, their models do not include the voltage-gated Hodgkin-Huxley (HH) conductances responsible for spiking, so they do not establish *how quickly* populations of realistic HH-based spiking neurons might perform approximate the necessary mathematical transformation.

Updating p(s) given new outcomes o according to Eq. 2 describes *perception* as inference.

*Planning* corresponds to optimizing one’s plan π based on minimizing *expected* free energy—the integrated free energy expected starting now and accumulating into the future. Recall that planning implies an agent with temporal and counterfactual depth, which is our operational definition of consciousness in the context of active inference.

Minimizing free energy with respect to plans (aka policies) implies the following optimal belief about plans π (again bold-font **π** denotes probability distribution P(π)):

(3) **π** = σ(-**F** – **γ** · **G**)

where **F** is the free energy of past outcomes averaged over plans:

$$F=\pi \cdot \sum_{t}^{T}F_{\pi t}$$

in which

**F**_πt_ = **s**_πt_ · (ln(**s**_πt_) - ln(**A**) · o_t_ - ln(**B**_πt_) · **s**_πt-1_).

Note that “the decomposition of [**F**] into a sum over time is due to the implicit mean-field approximation that assumes we can factorize the approximate posterior into a product of factors…In logarithmic form, this becomes a sum…” [100](p74). We will revisit this mean-field approximation below.

**γ** (formula not shown for simplicity) is the distribution of the precision of (or confidence in) the estimated free energy, and **G** is the distribution *expected* free energy averaged over potential plans:

$$G=\pi \cdot \sum_{t}^{T}G_{\pi t}$$

where

**G**_πt_ = **o**_πt_ · (ln(**o**_πt_ - **U**_t_) + **s**_πt-1_ · **H**).

Here the vector **U**_t_ encodes prior preferences regarding outcomes, aka utility; and the vector **H** encodes the entropy (ambiguity, uncertainty) of the belief distribution over outcomes for each hidden state.

Upon adopting the variational gradient descent approximation, one can express the state and precision update rules in “biologically more plausible fashion” [46] in terms of predictions errors. That is, the expressions for *gradients* of free energy take the form of prediction errors: differences between expected and observed values. For example,

(4) $$\frac{d}{\mathrm{dt}}\ln\left(s_{\pi t}\right)=\frac{d}{\mathrm{dln}(s)}\left(s_{\pi t}\right)\cdot \epsilon_{\pi t}$$

where **ε**_πt_, the state prediction error derived as the gradient of the free energy with respect to changes in states, takes the following form:

**ε**_πt_= (ln(**A**)·o_t_+ln(**B**_πt-1_)·**s**_πt-1_+ln(**B**_πt_)·**s**_πt+1_)-ln(**s**_πt_).

As above, “the postsynaptic depolarization caused by afferent input [is] interpreted in terms of free energy gradients (i.e., state prediction errors) that are linear mixtures of firing rates in other neurons (or populations)” [46]. Thus, again, linear combinations—weighted sums of firing rates—are a crucial ingredient for implementing active inference.

It is also critical to note that the “instantaneous” differential formulation in Eq. 4 does not evade the requirement for *discrete* finite-duration cycles of perceptual inference that we noted above. In this particular process model [46], Eq. 4 is supplemented by discrete steps of inference implemented “by hand” every 250 ms. Without those it would not be a model of temporally-deep, conscious active inference.

This form of the update rule is being called “biologically more plausible” because the product might be implemented by known Hebbian coincidence detection mechanisms believed to underlie long-term memory [20], [93]. Thus, synaptic weights between pairs of neurons could encode conditional probabilities of model parameters computed by the active inference algorithm [24]. If we assume presynaptic firing rates are transformed linearly into postsynaptic depolarizations proportional to synaptic weight, and we further assume that postsynaptic depolarizations from all synapses sum linearly, then we have a mechanism for computing *some of* the linear mixtures of rates we need. However, we will see below that these assumptions are not valid for realistic classical neural models.

Similarly, the prediction error term might be computed as differences between activities of prediction neurons and sensory neurons, which might be implemented by the neural architecture shown in Fig. 1b—understanding subtraction as a special case of addition.

Clearly the matrix multiplication operations in (2), (3) above require the ability to calculate weighted linear sums of firing rates (Table 1 row b). Addition is necessary in general for calculating expectations values of model parameters and policies, as well as for calculation of marginal probability distributions. This summing operation is mandatory for path integration and model averaging (rows c and d) for determining the agent’s best course of action (i.e., policy) going forward. It is also required for flexible readout of results from neural populations (row h)[25]. Its biophysical plausibility is supported by “realistic” conductance-based integrate-and-fire models in [85], [92], to be discussed further in Section 5.

If an agent has the capacity to add probabilities (row b), then in principle it has the capacity for path integration (row c, considering all possible courses of action under a particular model of the world) and model averaging (row d, performing a weighted average over possible models of the world). The path integral is indispensable, and model averaging is superior to simply choosing a best model [40].

Then, to make the path integral over possible future trajectories *tractable* [100], [55], [54], the “mean-field” approximation that we mentioned above (Table 1, row e) is introduced in which distributions over model parameters are replaced by their expected (mean) values. This amounts to a Markovian assumption that sensory-updated posterior belief distributions representing different times steps are statistically independent. Thus, their joint probabilities may be factored into simple products of separable variables [100], [46], [48], [54], [95]. In many deterministic models, neural activities represent repeated *time derivatives* of the variable whose distribution is being represented (instead of means and variances as in the simplest case)[99], [47], [49]. Such a representation can capture *proximate* temporal dependencies, but not entire trajectories.

Please note the importance of this mean-field approximation, as we will have occasion to revisit it below in 5, 6, 7. Related mean-field approximations treat different causes, inputs, or time scales as statistically independent [20], [48]. Like the gradient descent approximation, the mean-field approximation is not guaranteed to reach an optimal solution, in the sense of a global minimum of the variational free energy. These approximations have some face biological plausibility because neural activities representing model variables for different causes could be decorrelated by recurrent inhibitory connections among neurons representing independent time steps or causes [110], [85].

Matrix multiplication, as well as factoring probabilities according to the mean-field approximation, both imply a need for multiplication of probabilities or distributions (Table 1 row f), which is complementary to addition. In the neural process model we considered above, the necessary multiplications are converted into sums in terms of log-probability membrane potentials, or implemented as synaptic weights times presynaptic firing rates.

Divisive normalization (Table 1 row g) by some global measure of population activity is necessary to maintain a consistent interpretation of the individual neural activities in terms of probabilities. This appears straightforward to implement via recurrent connections from inhibitory neurons [110], [30].

## 4 The path integral formulation of active inference

The inferential planning governed by free energy minimization outlined above turns out to be mathematically equivalent to physical dynamics governed by Hamilton’s Least Action Principle (LAP). “A profound consequence [of the free energy principle] is that living organisms behave according to Hamilton’s principle of Least Action: they follow a path of least resistance until they reach a steady state (or a trajectory of states)…As such, Active Inference *is* Hamiltonian physics applied to a certain kind of system (systems that feature a Markov blanket)”(p54–55)[100].

In turn the principle of least action may be formulated in terms of a *path integral*, which is a procedure used to determine the probability of a transition by summing the probability amplitudes of all future trajectories whose endpoints are consistent with this transition. As we will see in Section 6, it appears to be the function used by nature to compute an *optimal* next step to actualize, in the sense of minimizing the *physical* action. Note that action is a quantity integrated over time during each potential trajectory of the system, like the expected free energy computed by a conscious agent. “This formulation expresses the probability of a path in terms of the action associated with a trajectory. It says that the surprisal (i.e., negative log probability) of a path (i.e., action) is the surprisal accumulated along its trajectory, based on the difference between the path’s motion and the flow expected at each point in state-space” [44].

This cumulative surprisal is called the action *S*:

*S*(s(t)) = -ln p(s(t))

and its evolution is governed by the integral:

*S*(s(t))= ∫*L*(s’,s,*t*)·*dt*

from zero to time t, in terms of the Lagrangian *L* given by:

$$L\left(s,s’\right)=\frac{1}{4\Gamma }s’\mathrm{\cdot s}’-\frac{1}{2\Gamma }\mathrm{f\cdot s}’+\frac{1}{h}V(s)$$

where s’ denotes $\frac{\mathrm{ds}}{\mathrm{dt}}$. Here f is the deterministic gradient-descent flow term in the continuous time expression of inference dynamics (essentially the right-hand-side of Eq. 4), and Γ is the half-width of the Gaussian noise assumed in the dynamics. h is Planck’s constant (set = 1 for a classical treatment) and V(s) is a “Schrödinger potential.”

In the classical limit with zero noise (Γ = 0), “the classical path is the most likely path that can be described with a variational principle of least action:

s(t) = arg min_s(t)_*S*(s(t))

This means the most likely path minimizes action” [44].

How do we see the connection to the free energy formulation above? In a temporally deep agent capable of planning, “the most likely course of autonomous behavior minimizes expected free energy” [44]:

G(s(t)) α *S*(s(t)|π)

**s**(t) = arg min_s(t)_*S*(s(t)|π) ≈ arg min_s(t)_G(s(t))

Note the exactness of the first equality in terms of the action, and the approximation when using expected free energy. This points to the fact that the path integral formulation can be indispensable in some cases where the gradient flow formulation does not apply (p115)[44]. Thus, the sum of G over time under different potential policies in Eq. 3 above can be understood as path integration (Table 1, row c), in which each potential plan is weighted according to its likelihood of being helpful. From this formulation we see that the active inference path integral is not just analogous to the physical path integral; it is mathematically equivalent.

## 5 Challenges for fast real-time inference

### 5.1 Combinatorial explosion problem

The core of the conscious active inference algorithm we outlined in Section 3 is the path integral that considers potential courses of action. The rub is that every possible action must be considered at every time step for every model, for every variable or cause in the model, and the number of time steps and possible trajectories could be very large. It appears that we require independent neural populations for every combination:

N_pops_ = N_causevars_ x N_controlvars_ x N_plans_ x N_models_ x N_params_

Here N_plans_ = N_timesteps_ x N_actionsperstep_, i.e., the number of possible plans for the upcoming time period. That is, each action control variable in a model can take N_actionsperstep_ possible values at each time step. N_plans_ is the number of “paths” in the path integral for each model, and each path must take into account multiple relevant variables each represented by a separate neural population, for each time step. For example, a simple model of a behavioral context with four potential target locations and four time steps in a trial required 16 neural populations to implement [98].

For real-time inference, all these potential paths need to be evaluated in the path integral sum, in about 200 ms. This is a very challenging computational constraint.

To assess the biological plausibility of this kind of scheme, we need some estimate of the number of available neurons for each represented variable, and some behavioral estimate of how many possibilities humans or other animals consider. Bastos et al., [9] mapped the active inference algorithm onto the microcircuit that is replicated throughout cortex (Fig. 1b). They explain: “The notion of a canonical microcircuit implicitly assumes that each circuit is distinct from its neighbors, which could presumably carry out computations in parallel. Therefore, the canonical microcircuit specifies the spatial scale over which processing is integrated. The most likely candidate for this spatial scale is the cortical column, which can vary over three orders of magnitude between minicolumns, columns, and hypercolumns.” The smallest cortical modules are the minicolumns, which span 50–60 µm [9] and typically contain about 110 neurons, but this may vary by up to a factor of three in different brain areas and species [70]. A hypercolumn as defined by Hubel and Wiesel contains modules for every possible value of some receptive field property such as orientation preference (typically 50–100), and typically spans 500–1000 µm [9].

### 5.2 Linear combinations of firing rates

The operation of addition is obviously critical for computing the path integral, which is after all a sum. How does one compute linear sums with neurons? And how *fast* can it be done with realistic neural models?

To approach these issues systematically, we must distinguish: (i) experimental evidence of what neurons can do, from (ii) idealized models of what neurons do, from (iii) realistic neural models based on the voltage-gated Hodgkin-Huxley (HH) dynamics. The HH conductances are the classical mechanistic basis of the action potential. To establish that classical neurons can perform a certain operation (summing firing rates) in a certain time (200 ms), requires single-neuron models that include the HH dynamics of action potential generation.

Consulting *Biophysics of Computation* [80], we see (p422) that the firing rate input-output relationship for a model pyramidal cell [11] is not even approximately linear in the range up to and around 60 Hz. These calculations include the presence of voltage-gated dendritic conductances and other synaptic nonlinearities. Moreover, neurons in sensory cortical areas do operate in this frequency range [23], [22]. So: in classical models, linear sums of firing rates are not the natural result of convergent neural projections.

The same textbook notes repeatedly that synaptic nonlinearities abound, but “almost all” models assume linearity of summation of synaptic inputs (p345). The assumption of linear summation of synaptic inputs has some experimental support from a study of cultured pyramidal neurons [17]. However, this putative linearity pertains only to subthreshold voltages, not the *firing rates* that are assumed to be the neural outputs in process models. The firing rate input-output relationship cited above shows that firing rates do not add linearly, even if synaptic potentials do. Moreover, this experimental result has been superseded by results in a more physiological slice preparation, showing complex but systematic nonlinear interactions in the dendrites of pyramidal cells [108]. Synaptic potentials probably *don’t* add linearly *in vivo*.

On the other hand, the textbook notes that sometimes real neurons exhibit “surprising linearity” (p476). For example, [28], [29] report a “remarkably linear” relation between input current and output *firing rate* in medial vestibular neurons. Why is such a result “surprising” and “remarkable”? Because “the fact that the spike generation is linear would appear to provide a significant challenge from the point of view of the neuron…. How active, nonlinear conductances are coordinated to produce a linear spike generator in any neuron remains unknown” [29]. Based on our review of the more recent literature below, we will conclude that this statement remains true in 2025.

We have other clear examples of linear summation by real neurons. The neuronal population vector [5], [56], [57], [60], [61], [58], [59], [64], [65], [79], [82], [83], [84] that encodes movement direction in multiple parts of the brain and nervous system of animals from crickets to humans, is linear. Similarly, perceptual color space appears to be represented by a population code representing a linear vector space, at least at some stages of the neural processing [12].

The question is, how can we get realistic, highly nonlinear neurons to implement such linear representations and computations, in about 200 ms? We are not questioning whether classical neurons can be tuned to approximate linear sums of firing rates given unlimited time or numbers of neurons. We are questioning whether realistic HH-based neurons can perform the necessary sums for path integration *quickly enough* to account for our perceptual inferences and behaviors that are updated on sub-second time scales.

### 5.3 Implementing linear summation requires too many trials for real-time inference

Ma et al. [85] showed linear summation of inputs by a network of 1260 conductance-based spiking neurons (Fig. 2B and C in that paper). To achieve this result, they tuned the network synaptic weights and *averaged over 1008 individual trials.* Thus, we can estimate that on the order of *one million* neurons (1260 ×1008) would be needed to achieve comparable linearity in a single trial—as required if an animal is to use statistical inference in real time. This number of neurons is on the upper end of the range for a whole macrocolumn, which is supposed to cover all possible values of a feature, not just one. Given the impressive match between the active inference model and neuroanatomy and physiology reviewed in Section 2, this consideration of numbers raises the question of whether “realistic” conductance-based spiking units can account for real observed perceptual behavior.

The “realism” of the models is also dubious, because only synaptic and adaptation currents are included in the model, not the voltage-gated channels responsible for spiking in real neurons. This same limitation applies to a model included in the supplementary information of [92]. That model used 100 neurons whose activity was sampled for *2000 s*, to achieve a linear regime in the network’s input-output relation. Again, this calls into question whether such a mechanism could account for perceptual inference happening on sub-second time scales. Similarly, real dendrites manifest complex nonlinear dynamics not captured in these simple models [108], [120]. Moreover, as we discuss further below, current models rely on attractor dynamics or duration sampling to estimate probability distributions, which are probably too slow to account for perceptual inference and real-time control.

Now, a hierarchical architecture might substantially alleviate this problem of numbers by allowing higher-level variables to “cover,” i.e., stand in for, many lower-level possibilities. However, this potential savings in terms of the necessary number of neurons is limited by varied evidence that we include much more than just higher-level summary information in our ongoing predictive processing. For example, speech perception involves implicit maintenance of acoustic and phonological details over dozens of syllables in order to successfully disambiguate inputs [116], [16], [32], [66], [76], [81]. In the active inference context, this means the path integral involves many possibilities, not just a few high-level possibilities. Similar considerations apply to speech production, and rationally optimized motor control in general [117], [118].

### 5.4 Classical neural attractors are slow

Moreover, others have noted that “convergence into attracting steady states” is too slow to account for the speed of real-time perceptual inference. This dynamical mechanism is assumed in almost all active inference process models whether deterministic or stochastic. Fekete et al. [34] point out that attractor states tend to be destabilized both by (a) different conduction delays among cortical areas, and (b) rapidly changing sensory input. It thus appears implausible that realistic networks could settle into quasi-stable activity patterns (attractor states) fast enough for real-time perceptual inference. Empirically, [127] demonstrated attractor dynamics in rats’ neural representations of different spatial environments. Attractor states in new environments equilibrated remarkably slowly, on the order of tens of seconds.

Likewise, [120] identify a “spiking bottleneck” for attempting to estimate continuous analog values with spiking neurons.

### 5.5 Neural stochasticity

Another limitation with respect to providing a plausible implementation of linear summation for deterministic process models, is that these conductance-based models are *not* purely deterministic. In order to achieve tolerable linear summation in an average over many trials, they assumed idealized Poisson stochasticity in the sensory inputs. If we model neurons as deterministic conductance-based charge integrators with deterministic synaptic transmission (like [85]), then all variability in neuronal activities arises from variability in inputs (c.f. Deneve [24]).

However, real neurons exhibit intrinsic stochasticity (Table 1 row i), primarily arising from ion channel noise and probabilistic neurotransmitter release at synapses, as supported by empirical observations [38], [4]. Importantly, this intrinsic variability is distinct from and cannot be fully accounted for by stimulus-driven variability alone. Similarly, the “unreliability” of synaptic transmission is more than can be explained in terms of inputs or recent history of activity [13], [14], [3], [74].

The above estimates of the necessary numbers of neurons for implementing real-time temporally-deep active inference are heuristic, and based on only two conductance-based modelling studies from over a decade ago. They are not conclusive. *That is our point*. To our knowledge, these two limited conductance-based modelling studies are the entire basis for the—mostly implicit—assumption that classical neurons obeying deterministic Hodgkin-Huxley dynamics could implement the sophisticated and complex active inference algorithms described in the large literature on active inference process models. This question of the required numbers of neurons for fast linear summation applies to both the deterministic parametric and sampling approaches (to be discussed further below) to representing probability distributions with neural activities.

Taking stock, we have identified several computational mechanisms assumed in parametric process models that are not well-supported by realistic neural models.

1. There is the requirement for dual probability and log-probability representations (as in (2), (3), (4) above), which are assumed to be fulfilled by firing rates and dendritic potentials respectively**,** but this conjecture is supported by a single empirical study and no modeling to our knowledge.
2. There is the requirement for robust linear addition, supported by two idealized conductance-based models. These models appear to require too many neurons, or too much time for perceptual inference on sub-second time scales. We are not aware of any HH-based modeling studies demonstrating how the heuristic considerations above may be evaded.
3. Attractor dynamics appear too slow for fast perceptual inference and motor control.
4. Real neurons exhibit *stochasticity* that is not accounted for by sensory inputs, but modeling studies have not established that realistic conductance-based spiking neurons can perform the continuous deterministic computations required by parametric process models.

### 5.6 Neural dynamics as stochastic sampling

Another way to finesse the potential combinatorial explosion discussed above is by adopting a *sampling* approach to inference ([129], [15], [39], [67], [92]; Dong [27]; [75], [107]). This approach may be distinguished from the deterministic parameter estimation or “sufficient statistics” approach we discussed in the previous subsection. In the sampling approach, neural populations are assumed to encode probability distributions by integrating over repeated (or a single) samples from the relevant (approximate) distribution, rather than by encoding parameters that refer to an implicit assumed parametric form of the relevant distribution.

Fiser et al. opined that having populations implicitly refer to a specific parametric model appears biologically implausible, whereas sampling directly from a non-parametric empirically estimated distribution appears less arbitrary [39]. This is debatable because a Gaussian distribution arguably could occur naturally due to the central limit theorem—if available time and numbers of neurons are sufficient. Similarly, another common parametric approach is to describe a probability distribution in terms of increasing derivatives of the represented variable. These might conceivably be implemented by neurons computing temporal differences. On the other hand, varied behavioral evidence strongly supports that humans and other animals often base decisions on relatively few samples from a complex multidimensional distribution [101], [123], [129], [18], [30], [62], [94].

### 5.7 Sources of neural stochasticity

If we do pursue the sampling approach, we must account for how realistic neurons could implement such stochastic sampling from a complex and varying distribution. The intrinsic stochasticity of synaptic transmission that we noted above may provide a mechanism for probabilistic sampling of whole activity patterns, that we need for implementing active inference with neurons. Even though the voltage dynamics of neurons are understood according to the deterministic Hodgkin-Huxley equations, the required stochastic sampling (Table 1, row i) has some face validity as a biophysically plausible mechanism because synaptic transmission is probabilistic [13], [14], [3], [74].

The synaptic stochasticity idea has been realized in impressive form by [89], who showed that synaptic stochasticity is sufficient to implement “complete” inference for learning. In particular, synaptic failures allow the network to explore alternative activation patterns representing distinct multidimensional possibilities stochastically, enabling gradient descent on a cost function. This is exciting, but excitement must be tempered by the realization that such a classical dynamical network attractor mechanism (a) requires at least hundreds of milliseconds to perform one sample, and (b) requires many samples to achieve an approximately optimal solution. This is why the proposed mechanism is limited to *learning* over longer time scales, but does not appear biologically realistic for perceptual inference or real-time motor control.

Similarly, [27] write “the view of stochastic sampling naturally accounts for the irregular firing and other response properties of neurons observed in the experiments. Although the idea of sampling-based inference is appealing, a critical concern is whether stochastic sampling is fast enough to match the rapid computation of the brain. For instance, the sampling trajectory of Gibbs sampling or Langevin sampling essentially performs random walks in local regions rather than the whole posterior space, which is too slow to be compatible with brain functions. Thus, it is crucial to explore whether neural circuits in the brain have the capacity of realizing sampling-based inference rapidly….”

### 5.8 Classical models require discontinuous “jumps” and non-Markovian irreversibility to speed sampling

Several studies have reported theoretical strategies for speeding sampling in classical neural models of inference ([1], [120], [69]; Dong, [27]). Ujfalussy et al. [120] explored how dendritic nonlinearities may enable linear sums of analog quantities in networks of spiking neurons, and [1] showed that networks of excitatory and inhibitory neurons exhibiting collective neural oscillations can speed sampling by an order of magnitude. These models don’t rely on slow convergence to attractor states for sampling, but on the other hand they don’t provide a realistic conductance-based model to show that realistic classical neurons can display the required stochasticity for fast sampling. In addition, these models and the ones discussed below rely on “duration sampling,” meaning that probabilities are represented by the relative durations spent in different neural activity patterns. Though not explicitly attractor representations, this duration sampling scheme may face the same issue as we identified for attractors: it may take too long to establish precise probabilistic representations if the brain has to wait for the network to spend “enough” time in each of several relevant activity states. Aitchison, Lengyel [1] also close their paper with an admission that the linear dynamics assumed in their model have not been demonstrated in mechanistic neural models.

The models analyzed in [69] require external input noise for their sampling stochasticity—which we have already seen is not realistic [38], [4]. However, they report the important finding that introducing irreversible non-Markovian dynamics appears necessary for fast sampling. “The fact that [non-reversible] networks are faster samplers is in line with recent machine learning studies on how non-reversible Markov chains can mix faster than their reversible counterparts. The construction of such Monte-Carlo algorithms has proven challenging, suggesting that the brain—if it does indeed use sampling-based representations—might have something yet to teach us about machine learning.”

Echeveste et al. [30]; Dong, [27] also found that abandoning Markovian reversibility allowed them to achieve faster sampling. Similarly, [15] argued that Gibbs sampling is biologically unrealistic, and showed that irreversible, indeterministic, and *discontinuous* “jump processes” are more faithful to the real dynamics of spiking neurons than continuous deterministic time-symmetric models that rely on input variability for stochasticity.

These studies collectively suggest that fast sampling requires abandoning idealized Langevin and Gibbs sampling so as to include discontinuous and *irreversible* “jumps” in the neural dynamics.

These approaches explicitly discard the Markovian mean-field assumption (Table 1, row e) that allows different time-steps to be treated by independent neural modules. Remember that the mean-field approximation was adopted to make the path-integral over possible plans *tractable* (by classical computation). We are abandoning that approximation to achieve biological plausibility in terms of sampling speed, but it appears that the cost is to eliminate the model’s biological plausibility in terms of problem tractability. Specifically, dropping the mean-field factorization of time-steps raises new doubts about the numbers of neurons needed, now that different time-steps in our plans cannot be treated as independent (which after all was not a realistic assumption in the first place, since each potentially useful policy involves a coordinated trajectory across time). Instead of rescuing the plausibility of classical approaches, these studies ([15], [69]; Dong, [27]) appear to doom them.

The non-Markovian probabilistic time evolution also suggests a quantum-like indivisible stochastic process that we will discuss further below in Sections 6 and 7, and in the companion paper following this one.

Above we noted that potential sources of intrinsic neuronal stochasticity include ion channel noise and probabilistic neurotransmitter release at synaptic terminals. In this connection, [63] have recently shown that the FitzHugh-Nagumo spiking neuron model plus noise, which appears to capture real spiking dynamics, is equivalent to the deterministic quantum Schrödinger equation for many variables with the addition of a stochastic term implicitly representing wavefunction collapse (to be introduced in Section 4 below). Remarkably, by comparing their model’s quantum parameter “h” to experimental data regarding single-neuron spiking, they conclude: “this lends considerable empirical support for the hypothesis that quantum-like effects play a non-negligible role at the level of neurons.”

*

The first four problems identified in our list above for deterministic models also apply to stochastic sampling models: the problem of fluent alternation between addition and multiplication, the problem of robust linear addition, and the problems of insufficient numbers of neurons or insufficient time. The issue of stochasticity is distinct but comparable for the stochastic models. Whereas deterministic models face the issue of demonstrating that a deterministic model is consistent with the stochasticity of real neurons, stochastic sampling models face the problem that our realistic description of neural membrane dynamics—the Hodgkin-Huxley equations—are deterministic.

**Fig. 3:**
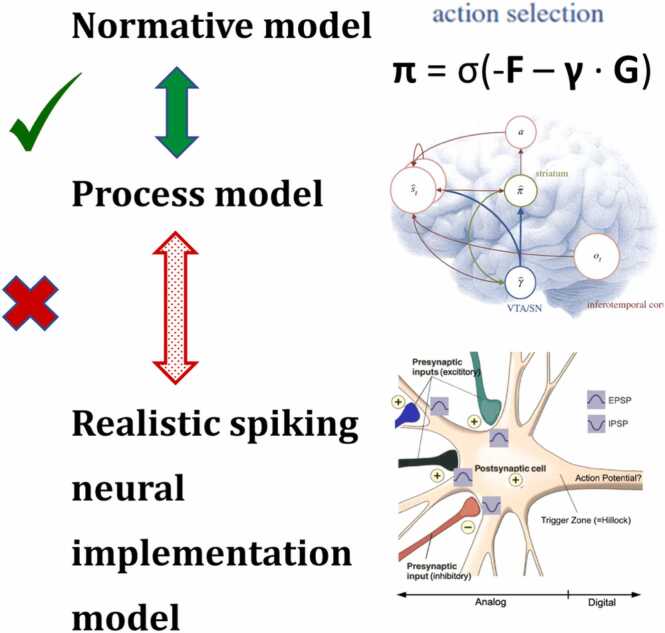
Schematic summary of the argument so far. The *green arrow and check-mark* indicate the admirable (though *coarse*) match between the variables of the active inference normative model (with approximations, *top row*) on the one hand, and neuroanatomy and neurophysiology (the neural process model, *middle row*) on the other. The *red arrow and “X”* indicate the failure (so far) of realistic conductance-based spiking neural models to account for the brain’s demonstrated probabilistic processing. In particular, realistic *numbers of neurons* appear insufficient for linear summation by realistic neural populations; and convergence to metastable attractor states appears to *take too long* to account for real-time perceptual inference and motor control. Brain image reproduced from Friston et al. [54], Frontiers in Human Neuroscience, under the terms of the Creative Commons Attribution License (CC BY).

Thus, detailed theoretical considerations raise doubts about the plausibility of current network models of either flavor. This argument is summarized schematically in Fig. 3. Crucially, we are not arguing that the ubiquitous use of idealizations such as firing rate models, or linear matrix multiplication to represent synaptic interactions, are generally invalid. We are simply pointing out that it has not been established that realistic single-neuron models incorporating the HH spike-generation dynamics can robustly approximate the daunting combinatorial sums of the path integral *in only 200 ms*.

We don’t think these considerations *rule out* a possible implementation of realistic temporally deep active inference with realistic numbers of realistic classical neurons. We raised them to motivate considering an alternative, experimentally supported *quantum* implementation. We briefly review quantum physics in the next Section, and then explain our proposal that path integration performed by a quantum system would provide a natural implementation of conscious active inference. We reserve discussion of experimental evidence concerning the specific biological substrate of quantum active inference for the companion paper that follows this one (conscious active inference II).

## 6 Quantum physics primer

“Quantum physics” (QP) is a generic term that includes “quantum mechanics” (QM) and “quantum field theory” (QFT), as well as more speculative models like string theory. We will focus here on QM and QFT. QM was historically the first quantum theory. The essential quantum features of indeterminacy and nonlocal coordination are already present in this primal version of the theory. However, QM is limited to non-relativistic physics. Moreover, particles are treated quantum mechanically while fields like the electric field are treated classically. In contrast, QFT may be thought of as a comprehensively quantum theory of matter and energy. It is fully compatible with special relativity, incorporates spin (which is intimately related to special relativity), and treats “force” fields with the same quantum principles as “matter” particles like electrons. For example, the electromagnetic interaction is understood in terms of exchanges of “photons,” which are irreducible “chunks” of electromagnetic energy (i.e., minimal excitations of the electromagnetic field). QFT manifests even more amazing classically-impossible phenomena of *coordination*, such as superconductivity, in which electrons conspire to pass through solid metal with zero resistance. The Standard Model of elementary particle physics is a QFT.

Quantum theories describe *quantum states* or *wavefunctions* which determine the probabilities of different potential measurement outcomes. In a quantum system, when more than one outcome is possible, states are said to be *superpositions* of the different measurement outcomes. Importantly, these superpositions are perfectly *linear* sums of distinct possibilities. Textbook quantum theories have two parts to their description of the evolution of states over time. The first is a local deterministic evolution described by a differential equation like the Schrödinger Equation. The second is an indeterministic and discontinuous (i.e., discrete) change in the quantum state that occurs at the moment of a *measurement*—the so-called collapse of the wavefunction.

This irreducible indeterminacy is one essential difference between quantum and classical physics. The other is the instantaneous non-local *coordination* among distant parts of a system when any of them are subjected to a measurement. *Entanglement* is the technical term for the nonclassical correlations among different particles or spatially distributed parts of a system. However, it is important to understand that entanglement arises locally according to the local deterministic Schrödinger-like dynamical evolution, and the non-local, holistic influences only manifest upon measurement. This holism is objective and experimentally verified over decades, resulting in the 2020 Nobel Prize [90]. Entanglement is the key quantum property that enables this holism and confers the advantages of quantum computation over classical computation (for certain applications).

Though QM and QFT rely on the concept of a measurement in order to produce meaningful predictions from the theory, neither provides a definition of what physical interaction or process actually constitutes a “measurement.” This is known as the Measurement Problem [124]. In this first of two companion papers, we take an agnostic attitude regarding this issue, but in the second paper we will focus on a particular approach to solving the MP.

### 6.1 Active inference predicts quantum biology and quantum consciousness

As a first step toward considering a quantum physical implementation of active inference, we note that [37] formulated active inference for quantum systems, and *derived* that quantum entanglement and quantum computation *must* be involved in consciousness and widespread in biology. Thus, the conclusion that active inference must exploit non-trivial quantum effects derives from the active inference theory itself. This remarkable result appears to have gone widely unnoticed.

### 6.2 The path integral formulation

is derived from the Least Action Principle (LAP) that we noted above is equivalent to the free energy principle underlying active inference. It is arguably the most fundamental formulation of QP [102], [35], [77]. Most scientists including physicists learn QM in an alternative formulation known as the operator formalism, because it is less mathematically demanding (among other, valid, reasons). Unfortunately, this means many are unaware that the path integral formulation is fundamental. For instance, many textbooks introduce the non-relativistic Schrödinger equation as a *postulate* of QM, implying that it is not derivable from more general first principles, but rather that it *is* a general first principle. This Schrödinger equation is not general, as can be seen by noting that it is not relativistic and does not describe particles with spin. In contrast, the path integral formulation bases all of quantum physics on two relatively simple and intuitive postulates [77]. The familiar Schrödinger equation can be derived from these in the non-relativistic limit [102], [77]. The path integral formalism also provides a convenient relativistic quantum description of complex systems with constraints, and allows for elegant translation among other formalisms. Several more technical advantages of the formalism are described in [102], [77]. Concretely, the formalism gives a prescription for calculating transition probabilities between any pair of states over time, based on the LAP that minimizes the physical action.

It does so in terms of an integral over every enumerable trajectory between the initial and final state. A measurement is assumed to occur at the later time, which will give definite results according to the calculated probabilities for each outcome. Thus, although the path integral function represents probability amplitudes of possible outcomes, the physical system actualizes one of those possibilities upon measurement, in accordance with the LAP. As always in quantum physics, the summation of the probability amplitudes for all the trajectories is perfectly linear.

Specifically, the transition function representing the probability amplitude for a transition from position or state a to state b is given by [102], [35], [77]:

$$K\left(b,\;a\right)=k\cdot \sum_{t_{a}}^{t_{b}}e^{\frac{\mathrm{iS}}{h}}=k\cdot \int \mathrm{Dx}\cdot e^{\frac{\mathrm{iS}}{h}}$$

Here the capital ∑ represents the quantum path integral: a grand sum of the integrated exponential phase factors along *every conceivable whole path* from a to b. In the exponent, *i* is the square root of negative 1, and *h* is Planck’s constant divided by 2π. Small k is a constant.

The *S* in the exponential phase factor is the physical action given by the integral of the Lagrangian *L* over time from the time of initial state a to the time of final state b:

*S*[*b*,*a*]= ∫*L*(*x*’,*x*,*t*)·*dt*

Transition probabilities are calculated as the complex *square* of the probability amplitude *K(b, a)*. The equality on the right shows the sum over paths converted into a continuous *functional* integration represented by Dx.

These deceptively simple postulates encompass all physics according to quantum field theory. They describe a universal dynamic that considers potential physical actions, and chooses one to actualize by minimizing—*optimizing*—the physical action.

The Lagrangian here is analogous to the free energy in the free energy formulation of active inference, in that it is to be minimized. Its specific form depends on details of the physical system under consideration, expressed in terms of generalized coordinates *x*, *x’*, and *t*. In general, it is the system kinetic energy T minus its potential energy U:

*L = T – U*

The action is the integral of the Lagrangian over time, as we saw in Section 4. We also saw its direct relationship to *expected* free energy calculated by conscious agents to determine their immediate plans, where expected free energy is also an integral of possible paths over time. The potential function U(x) in the Lagrangian plays the role of **U**_t_ in Eq. 3 describing conscious real-time planning above. For example, a single massive particle in a field with potential U(x) is (typically) guided by the field to move to regions with low potential. This is analogous to how the **U**_t_ vector encodes preferred regions of state space: the goals and rewards that motivate an agent’s behavior in active inference.

As we reviewed in Section 4, the free energy minimization of active inference is mathematically equivalent to the least action formulation of physical dynamics [100], [44]. Under the Least Action Principle (LAP), a physical system’s trajectory over time minimizes the physical action. It is as if the system considers every possible future trajectory in the path integral and calculates one that will minimize (or “optimize”) the action over time.

The path integral itself outputs probability amplitudes, not a determinate single path. But the measurement, which cannot be omitted, selects a single next step on a trajectory, on the basis of the relevant probability amplitudes, so as to optimize the action. *Thus, the quantum path integral is mathematically equivalent to the integral by a conscious agent in active inference weighing potential plans to decide on an optimal one.*

However, in classical physics this *integral* point of view is redundant and dispensable, because the dynamics are completely specified by *differential* equations without any need for integrating over different possible future trajectories. The system simply follows “the path of least resistance.” In contrast, the holistic integral point of view is mandatory in quantum physics. As such, real quantum dynamics involve an indivisible stochastic process [6], [7], which violates the assumption of statistically independent time-steps implicit in the Markovian mean-field approximation inherent in many of the proposed classical implementations of active inference.

### 6.3 The quantum path integral incorporates non-classical paths

This is a fundamental difference between classical and quantum physics. The fact that the quantum path integral incorporates non-classical paths—indeed all conceivable paths—is the *basis of the advantages of quantum computation* over classical computation. In terms of conscious active inference, this difference is the basis for the quantum system’s ability to consider every potential path to find an optimum.

This is how a quantum system could solve the combinatorial explosion problem we noted above. The quantum path integral automatically incorporates every *combination* of potential future states. Moreover, it considers each potential future trajectory *as a whole*, in contrast to the classical models which only consider combinations of path elements that are statistically independent from each other at each time step (under the mean-field approximation).

This formal equivalence between conscious inference and quantum dynamics is the basis of our proposal that a quantum system can account for the computations required by active inference much more plausibly than a classical neural network. Again, this physical path integral is exactly analogous to the path integral a conscious agent in active inference uses to select among potential plans. We use the word “analogous” here because of the differing *interpretations* of the formalism in the active inference context as opposed to physics, but again, they are mathematically equivalent. That is why a quantum physical process is a plausible candidate for *implementing* the cognitive inference algorithm.

Our operational definition of consciousness in terms of counterfactual depth refers precisely to this rapid implicit planning function of the quantum path integral.

If sentient biological systems have evolved a way to map their “personal problems” onto the potential U(x) in a quantum system manifesting this universal optimization process, the quantum dynamic could naturally implement active inference [126].

In the next Section we explain the proposal in more detail, postponing discussion of supporting experimental evidence and another critical challenge—the problem of discrete perceptual cycles—until the companion paper (conscious active inference II).

## 7 The quantum path integral naturally implements conscious active inference

The quantum LAP implies that quantum physical systems minimize their action over time by performing a weighted sum of *possible* trajectories, to enact an optimal one upon measurement.

Each of the central challenges for the classical models of conscious active inference that we reviewed in detail in Section 5 is accomplished automatically by the quantum dynamic.

• The weighted sums (integrals) of probability amplitudes are exactly *linear* without any tuning of physiological parameters required (given physiology capable of supporting the quantum dynamics in the first place—to which we return in the companion paper).
• The potential combinatorial explosion of the path integral is defused because quantum systems perform this astounding integral over *all possibilities* as part of their fundamental dynamics.
• The stochasticity and discontinuity in the dynamics that were introduced *ad hoc* in the classical models, and which threatened to make their computation intractable, are irreducible features of quantum dynamics.

The quantum sum is fundamentally different from the LAP principle applied in classical physics, which is redundant with an instantaneous local description in terms of forces (i.e., gradient flows). The quantum case is different because quantum probability amplitudes are complex numbers, which means that different potential paths can interfere constructively *and destructively*, unlike classical probabilities. This is how a quantum associative memory can avoid the problem of interference among memories whose neural representations overlap [26], and how a quantum system can be more selective and intelligent in its integration over distinct possible futures.

So what is a quantum path integral good for? Like a classical path integral, the computation is suited for optimization problems with complex constraints. This is what the “variational calculus” of functional integration is applied to in general. The travelling salesman problem is a classic optimization problem, but many other problems can be formulated in terms of optimization. Many problems faced by biological agents take the form of optimizing trade-offs among competing needs and constraints. A simple biological example comparable to the travelling salesman problem is the problem of finding the most efficient path to a needed goal (food, say) through complex and changing terrain comprising varied obstacles and risks. Importantly, memories of past trips through the terrain enable much safer and more productive travel.

In reinforcement learning theory, animals are understood to optimize the accumulation of reward over time. And of course, in active inference agents are understood to minimize their expected free energy by an optimization process. Arguably, *all* problems can be formulated in these terms.

But in the quantum case, the trajectories that are summed are complete trajectories, not factorized proximate trajectory elements as in classical active inference approximations. Barandes [6] establishes that the quantum dynamics a *not* captured by Markovian processes with independent times steps (i.e., the mean-field approximation, Table 1 row e), which are the basis of most classical process models of active inference.

We suggest that considering complete trajectories is critical for exploiting episodic memories quickly “in the moment,” but is only feasible at biological time scales for a genuine quantum system.

Consider a predator navigating complex and uncertain terrain in pursuit of prey; or a prey animal seeking the fastest route to safety among varied obstacles and risks. The consciousness process produces perceptual interpretations and behavioral decisions on a moment-to-moment basis, by *implicitly* considering every possibility before consciously realizing an optimal one. Critically, this preconscious path integration refers to memories of preferred and repugnant outcomes at particular locations in the terrain or maze.

Let us focus on one crossroads in the maze. Many remembered episodes intersect with this place. Some ended well, others unhappily. Moreover, exiting the crossroads by a specific path sometimes ended well, sometimes badly. And the happy or unhappy ending may have depended on what happened near the *beginning* of each episode. This means the information about the best way to proceed *now* is not available to a classical mechanism that only has access to memories of *proximate* trajectory elements and their associated outcomes.

Classical approaches to this “temporal credit assignment problem” have been explored in classical neuroscience [20], but have not yet been incorporated into process models of active inference. Moreover, the classical approaches depend on maintaining a distinguishable neural “trace” of each past event that might be relevant now, so it appears implausible that they will account for our ability to consult *decades* of episodic memories on a moment’s notice.

More generally, quantum physics allow for the possibility of classically-impossible contextuality or semantic coordination effects, as has been documented in the literature on quantum cognition [109]. We will postpone further discussion of putative quantum cognition and the potential advantage of a quantum system for memory until the companion paper (conscious active inference II).

It must be appreciated that harnessing the power of the quantum path integral directly to solve optimization problems has not been the main approach of the quantum computation field thus far. Nevertheless, a number of approaches since the early 2000s have demonstrated quantum speedups on combinatorial optimization problems. These include quantum Markov chain algorithms [115], [128], the short path algorithm [68], and the quantum approximate optimization algorithm (QAOA)[114], [33], [72]. From a neuroscience perspective it is intriguing to note that the QAOA uses pairs of operators applied recursively; each pair is known as a “layer.”

Despite these demonstrations, mapping computational problems onto a quantum path integral instantiated by a concrete physical structure, especially in the context of neuroscience, is a new field. But we can be guided by the analogy between the quantum potential U(x) and the vector of preferences in active inference, **U**_t_.

### 7.1 A concrete neural model illustrates quantum active inference

An agent performing active inference is *like* a particle moving to the bottom of a complex potential U(x). A simple model [10] shows concretely how classical neural activities could “program” a hypothetical quantum state with an arbitrary potential U(x) so that the quantum dynamics would automatically find the minimum. In particular, [10] modeled eye-movements tracking a moving target. They simulated this quantum brain with a Schrödinger equation and compared performance to standard classical Kalman-filter-type algorithms. Although we are postponing further discussion of the MP until the companion paper (conscious active inference II), we must note here that this model *does* involve an implicit *measurement* step at which probability amplitudes for the target location are *squared* to produce actual useable probabilities to guide the simulated eye-tracking behavior.

The quantum model dramatically outperformed the classical Kalman filters, but perhaps more impressively, it introduced *discrete* jumps into the eye-movements that were absent in the classical simulations—but are present in real eye-tracking data. It appears the quantum model enabled classically impossible performance and better matched qualitative features of the data.

This is *prima facie* evidence that the quantum dynamics are responsible for the dramatic advantages of the quantum model. Under the path integral formulation, it implies that paths other than the classically most probable one were incorporated into the inference cycle. Recall that the Schrödinger equation that governed the quantum dynamics in this model can be derived from the quantum path integral, which we have shown is equivalent to conscious active inference. That is why this simple model represents a concrete instance of quantum active inference in a neural context.

Again, its advantage over the best classical models (Kalman filters) demonstrates that the quantum advantage—due to the quantum path integral—actually obtains in a realistic behavioral context, namely tracking a moving target with one’s eyes. This example shows concretely how quantum active inference could be implemented (at the algorithmic “process model” level) in a neural context, and demonstrates that it really does perform better than a classical model, as hypothesized.

We are not aware of newer developments superseding those results. The model is conceptually straightforward and might be generalizable to more complex movements and behaviors.

Note again that without the implicit collapse step, the model generates uninterpretable complex probability amplitudes that evolve continuously. Different theories of quantum physics (to be reviewed briefly in the companion paper) give different names or “interpretations” of this step, but the measurement or collapse events are necessary to manifest the performance advantage over classical models, and to reproduce the discontinuous jumps observed in real eye-tracking behavior.

The quantum eye-tracking model demonstrates concretely how a context of preferences can be directly encoded from classical neural activity patterns into a quantum system to produce optimal behaviors according to the active inference algorithm, using the quantum path integral as an inference engine.

### 7.2 Summary

We have argued that realistic classical neural models of predictive processing have failed to establish their biological plausibility, because they take too long for real-time inference. This motivated us to consider an alternative quantum implementation, based on the “profound” [100] correspondence between the computations required by active inference and the quantum path integral underlying physical dynamics.

### 7.3 Outlook

The functional importance of quantum entanglement in conscious agents has been *derived* from within the active inference formalism itself [37], so we expect more serious investigation of the role of quantum physics in biology and consciousness. In the companion paper following this one (conscious active inference II), we will review a quantum solution to the critical problem of discrete non-overlapping moments of perception, as well as recent experimental evidence for a specific quantum substrate of biological consciousness.

## CRediT authorship contribution statement

**Arjan Singh Puniani:** Writing – review & editing, Visualization, Resources, Investigation. **Wiest Michael:** Writing – review & editing, Writing – original draft, Visualization, Investigation, Conceptualization.

## Declaration of Competing Interest

The authors declare no competing financial interests.
